# Alcohol consumption, high-sensitivity C-reactive protein, and estimated glomerular filtration rate: tripartite predictors of 10-year cardiovascular risk progression in patients with type 2 diabetes mellitus after COVID-19

**DOI:** 10.3389/fendo.2025.1609144

**Published:** 2025-08-26

**Authors:** Yizhe Wang, Yu Duan, Yongcheng Zhang, Na Li, Ying Hu, Liping Gu, Yanfang Hou, Yuhang Ma

**Affiliations:** ^1^ University of Shanghai for Science and Technology, Shanghai, China; ^2^ Department of Endocrinology and Metabolism, Shanghai General Hospital, Shanghai Jiao Tong University School of Medicine, Shanghai, China; ^3^ Faculty of Engineering, University of New South Wales, Sydney, Australia

**Keywords:** type 2 diabetes, cardiovascular disease, COVID-19, SCORE2-diabetes, risk factors, cardiovascular-kidney-metabolic

## Abstract

**Objectives:**

To investigate the predictors influencing the advancement of 10-year cardiovascular disease (CVD) risk after infection with Corona Virus Disease 2019 (COVID-19) in type 2 diabetes patients, and to provide a theoretical basis for an early intervention program for the cardiovascular dimension of the cardiovascular-kidney-metabolic syndrome (CKM syndrome).

**Methods:**

A cohort of 378 individuals diagnosed with type 2 diabetes was analyzed retrospectively. The progression of 10-year CVD risk was characterized by an elevation in the 10-year CVD risk category, as determined by the SCORE2-Diabetes scoring system, in type 2 diabetic infected with COVID-19. Factors influencing 10-year CVD risk progression were evaluated through univariate and multivariate stepwise logistic regression. Nonlinear relationships between predictors and 10-year CVD progression were assessed using restricted cubic spline (RCS) analysis, subsequently followed by an analysis of threshold effects. Finally, the predictive performance of various factor combinations for 10-year CVD risk progression during the post-acute COVID-19 phase in type 2 diabetes mellitus cohorts was measured by area under roc curve (AUC).

**Results:**

After infection with COVID-19, 12.2% (n=46) experienced progression in their 10-year CVD risk category. In multifactorial stepwise logistic regression, alcohol consumption [odds ratio (OR) 2.10, 95% confidence interval (CI) 1.02-4.34], estimated glomerular filtration rate (eGFR) (OR 0.96, 95% CI 0.94-0.99) and high-sensitivity C-reactive protein (hs-CRP) (OR 1.33, 95% CI 1.13-1.57), were found to be significantly linked to the progression of 10-year CVD risk. Restricted cubic spline analysis (RCS) demonstrated a nonlinear correlation between hs-CRP and 10-year CVD risk progression with a threshold of 3.0 mg/L. 10-year CVD risk was significantly higher with increasing hs-CRP levels at hs-CRP < 3.0 mg/L (OR 2.28, 95% CI 1.48-3.55), and the two-stage model significantly superior to a single linear model (*P* = 0.028 for log-likelihood ratio). Among the different combinations of models for alcohol consumption, hs-CRP, and eGFR, the full model combination of all three had the best predictive effect (AUC = 0.749).

**Conclusion:**

Alcohol consumption and elevated hs-CRP were associated with increased cardiovascular risk progression, while higher eGFR levels were inversely associated with risk progression.

## 1 Introduction

According to the International Diabetes Federation, over 500 million people worldwide have diabetes mellitus, with type 2 diabetes mellitus (T2DM) accounting for more than 90% of cases (1). T2DM involves impaired pancreatic β-cell function (2), insulin resistance, and compensatory secretory insufficiency, ultimately leading to disrupted glucose homeostasis as the disease progresses. Notably, CVD accounts for 40% of China’s mortality rates (3–5), ranking as the top cause of death, and is the primary fatal complication in individuals with T2DM. Epidemiological data indicate that approximately 50% of diabetes-related deaths are attributable to CVD, with T2DM patients facing a twofold higher risk of cardiovascular mortality compared to healthy individuals (6, 7). It’s been shown through studies that individuals with T2DM have a lifetime risk of cardiovascular diseases that’s two to four times greater than those who don’t have diabetes, encompassing issues like heart disease, strokes, and heart failure (8, 9). Given the strong interplay between T2DM and CVD, the European Society of Cardiology (ESC) emphasized integrated risk assessment in its 2023 ESC Guidelines on Cardiovascular Disease Management in Diabetes (10), which suggests the use of the SCORE2-Diabetes (11) to estimate the 10-year risk of CVD in individuals with T2DM in Europe, and Fu et al. (12) confirmed the applicability of the SCORE2-Diabetes in Chinese individuals with T2DM by recalibrating it with a high-risk region rescaling factor, which improved its accuracy for this population. The guidelines also highlight the critical role of chronic kidney disease (CKD) in CVD management for individuals with T2DM (10), as nearly 40% of T2DM patients have comorbid CKD (13), and CKD progression exacerbates CVD susceptibility due to renal dysfunction (14). To reflect their systemic interactions, the concept of cardiovascular-kidney-metabolic (CKM) syndrome (15) has proposed. In CKM progression, the cardiovascular system of 10 serves as the primary target organ; CVD is not merely a consequence of metabolic dysregulation and renal injury but also accelerates global CKM deterioration, forming a vicious cycle (15).

The COVID-19 pandemic has further amplified health risks for T2DM patients. Abe et al. (16) found that diabetic individuals with COVID-19 infection experienced worse cardiovascular outcomes, such as a combination of cardiovascular events, acute heart failure, and the onset of atrial fibrillation. Koyama et al. (17) showed that individuals with diabetes who had been infected with COVID-19 experienced significantly greater post-acute cardiovascular risks compared to uninfected controls. According to Nandy et al. (18), individuals with diabetes were about 3.07 times more likely to have severe outcomes from COVID-19, those suffering from CVD faced a 4.5 times greater likelihood, and for those battling chronic kidney disease (CKD), the odds of a bad outcome after catching COVID-19 were a whopping 5.3 times greater. These effects likely involve multifactorial mechanisms such as direct viral injury and inflammatory storm activation (19, 20), potentially undermining the predictive validity of current SCORE2-Diabetes 10-year CVD risk models (11).Although integrated CKM management is widely endorsed, independent risk pathways within its components require further elucidation. Particularly in the context of COVID-19, identifying post-infection CVD progression risk factors in T2DM patients could provide pivotal evidence for precision stratification in CKM interventions.

This study aims to investigate risk factors for 10-year CVD risk progression in T2DM patients following COVID-19 infection, offering a theoretical foundation for early cardiovascular-directed interventions in CKM syndrome.

## 2 Materials and methods

### 2.1 Study population

**Figure 1:**
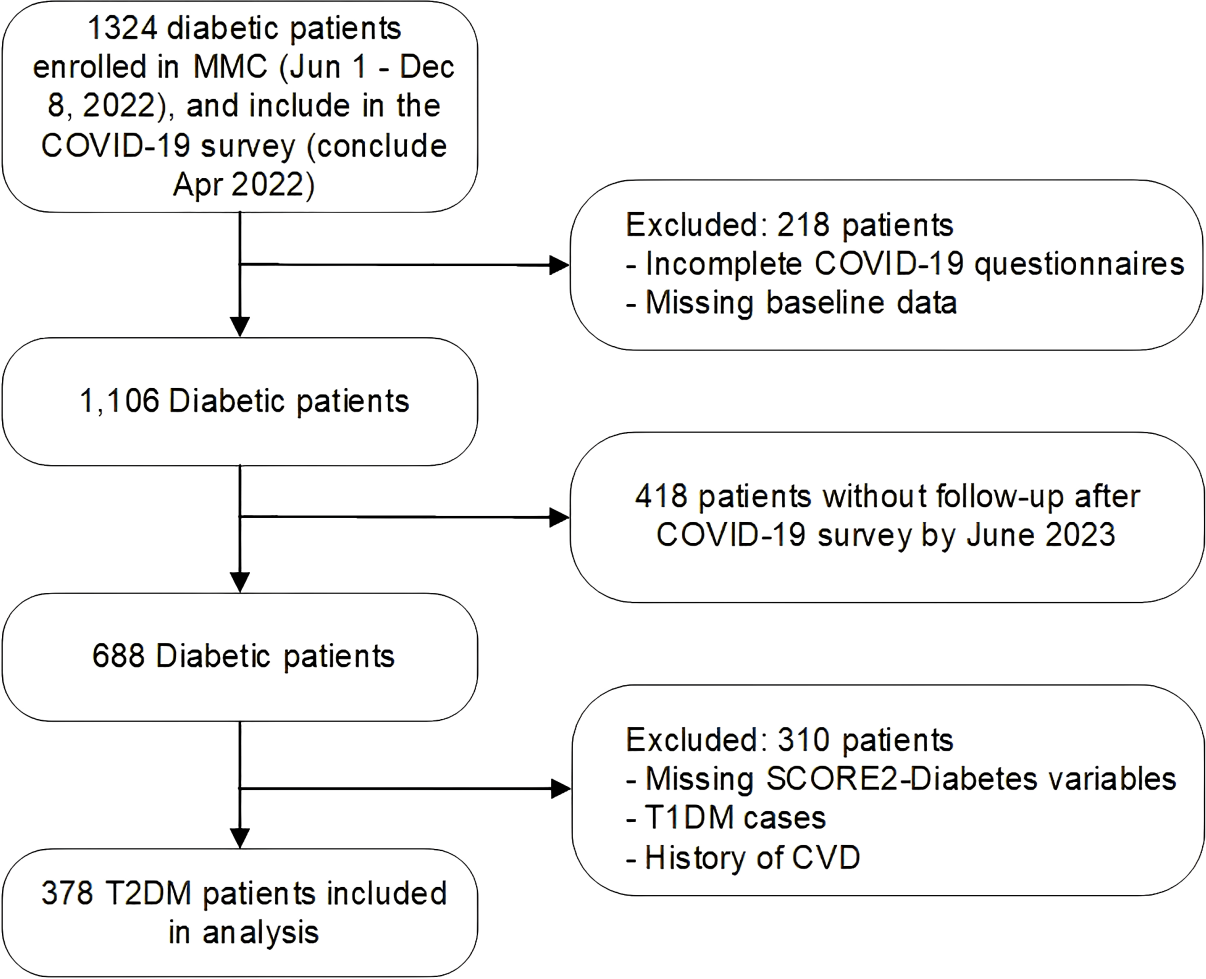
Flow chart of the study population.

We carried out a retrospective cohort analysis to evaluate 10-year CVD risk progression in T2DM patients after COVID-19 infection at the Metabolic Management Center (MMC) of Shanghai First People’s Hospital. A total of 1,324 patients with diabetes who joined MMC between June 1, 2022, and December 8, 2022, were included in the COVID-19 survey, which concluded in April 2022. This study used the most recent diabetes-related laboratory tests conducted within the six months prior to the patient’s COVID-19 infection date as baseline data, including sociodemographic characteristics, medical records, lifestyle behaviors, and laboratory test results. Among these, 218 patients were excluded due to incomplete COVID-19 questionnaires or missing baseline data. As of June 2023, 688 of the remaining 1,106 participants had completed follow-up diabetes-related tests post-pandemic. After excluding cases with missing data required for the SCORE2-Diabetes model, T1DM cases not classified as T2DM, and cases with a history of CVD, a total of 378 T2DM patients were included in the final analysis.(**Figure 1**).

Prior to implementation, the Ethics Committee at Shanghai General Hospital, part of Shanghai Jiao Tong University’s Medical School, had reviewed and sanctioned this research (No. 2017KY209). All methodologies conformed to the Declaration of Helsinki principles governing biomedical research ethics. Informed consent was obtained from all participants before enrolment.

### 2.2 Data collection

Data were gathered by the trainees following the MMC standard protocol (21), and according to the MMC standard operating procedures (21), spanning socio-demographic details, medical records, lifestyle behaviors, and laboratory tests such as glycosylated hemoglobin albumin (HbA1C), glycosylated serum albumin (GA), fasting glucose (FPG), fasting C-peptide (FC-P), as well as lipid profiles like total cholesterol (TC), triglycerides (TG), high-density lipoprotein cholesterol (HDL-C) and low-density lipoprotein cholesterol (LDL-C) (22). We also looked at kidney function markers such as blood urea nitrogen (BUN), serum creatinine (Scr) and morning urine albumin/creatinine (UACR) (23), and the inflammatory marker high-sensitivity C-reactive protein (hs-CRP) (24).

In our study, we defined the diagnosis of patients infected with COVID-19 as a positive test result after the viral gene detection by RT-PCR (25). Based on the questionnaire results collected in accordance with the MMC standard protocol (21), patients who drank alcohol weekly or almost weekly were defined as having a drinking habit, and smokers who smoked cigarettes daily or almost daily and less than once a day or less than 7 cigarettes a week were defined as having a smoking habit. Body mass index (BMI) was determined using the formula weight (kg)/height²(m²) (26). For insulin-treated patients, homeostasis model assessment of insulin resistance (HOMA-IR) and β-cell function (HOMA-β) were derived from FPG and FC-P levels (27). Estimated glomerular filtration rate (eGFR) was determined using the serum creatinine-based CKD-EPI equation developed by the U.S. Chronic Kidney Disease Epidemiology Collaborative Working Group in 2009 (28). Hs-CRP levels were stratified according to CDC/AHA guidelines (29): low-risk (<1.0 mg/L), medium-risk (1.0–3.0 mg/L), and high-risk (>3.0 mg/L).

### 2.3 Statistical analysis

Normally distributed data are expressed as the mean plus or minus the standard deviation (Mean ± SD), while non-normally distributed values are reported as the median with interquartile range (M(IQR)) (30). Categorical variables, on the other hand, are displayed as frequency counts alongside their corresponding percentages (30). One-way analysis of variance (ANOVA) (31) was employed to compare the means of normally distributed variables across the three CVD risk groups at baseline: low, intermediate, high, and very high risk, and independent samples t-tests (31) were conducted to assess the mean differences between the 10-year CVD risk progression group and the no progression group; for skewed distribution variables, the Kruskal-Wallis H-test (32) was applied to analyze the median differences in the data between multiple groups, and the Wilcoxon rank sum test (33) served to assess the median difference between the 10-year CVD risk progression and no progression groups. The chi-square test (34) was applied to compare categorical variables.

Univariate and multivariate stepwise logistic regression was used to further identify risk indicators associated with 10-year CVD risk progression after COVID-19 infection in T2DM patients. Variables with *P* < 0.20 in univariate analyses were entered into a multivariate logistic regression model using a stepwise selection procedure. Entry and exit criteria were set at *P* < 0.05 and *P* > 0.10, respectively. Logistic regression results are presented as odds ratio (OR) with 95% confidence interval (CI) and a two-sided *P*<0.05 was regarded as statistically significant (35). The risk indicators screened by multifactorial stepwise logistic regression were then stratified. The relationship with the 10-year CVD risk progression was characterized by nonlinearity, using restricted cubic spline (RCS) analysis, followed by threshold effects analysis. Finally, the performance of the predictors was evaluated using AUC. Statistical analyses were conducted using R version 4.4.2 software.

## 3 Results

A total of 378 T2DM patients infected with COVID-19 were included in this study. **Table 1** lists the baseline demographic and clinical characteristics of the participants grouped according to the SCORE2-Diabetes model. Of the 378 participants, 173 (45.8%) showed low risk, 123 (32.5%) were at intermediate risk, and 82 (21.7%) were at high or very high risk, in this study, the high and very high risk groups were combined into one group. The proportion of male rate, smoking rate, alcohol consumption rate, age, and age at diagnosis of diabetes (ADOD) all gradually increased and were significantly different among the three groups (*P* < 0.02), and SBP, HbA1c, FPG, GA all increased significantly with increasing risk class (*P* < 0.02). In contrast, FC-P and HOMA-β decreased significantly with decreasing risk class (*P* < 0.05). BUN, Scr and UACR all increased significantly with higher risk classes, whereas eGFR decreased significantly (*P* < 0.001).

**Table 1:** Comparison of general information at baseline for T2DM according to 10-year CVD risk grading.

Variable	Low risk (n = 173, 45.8%)	Moderate risk (n = 123, 32.5%)	High and very high risk (n = 82, 21.7%)	*P*-value
Male, n (%)	100 (57.8)	82 (66.7)	69 (84.1)	**<0.001**
Smoking, n (%)	15 (8.7)	44 (35.8)	45 (54.9)	**<0.001**
Alcohol consumption, n (%)	47 (27.2)	42 (34.1)	37 (45.1)	**0.017**
Age (years)	47.00 (44.00-52.00)	57.00 (51.00-62.00)	63.00 (59.00-66.00)	**<0.001**
ADOD (year)	43.00 (40.00-48.00)	49.00 (44.00-56.00)	53.00 (45.25-59.75)	**<0.001**
Height (cm)	167.00 (160.50-173.50)	169.50 (163.25-174.50)	168.50 (163.50-173.75)	0.266
Weight (kg)	70.72 ± 11.09	69.84 ± 11.03	69.85 ± 10.54	0.74
BMI (kg/m²)	25.00 (23.20-26.90)	24.10 (22.40-26.25)	23.95 (23.02-26.08)	0.073
SBP (mmHg)	127.06 ± 16.07	130.21 ± 15.10	133.35 ± 16.67	**0.011**
DBP (mmHg)	77.02 ± 10.70	76.87 ± 9.17	75.50 ± 10.68	0.518
HbA1C (%)	6.20 (5.80-6.70)	6.80 (6.00-7.30)	7.30 (6.70-8.35)	**<0.001**
GA (%)	13.94 (12.38-16.20)	15.58 (13.42-17.79)	17.45 (14.87-20.40)	**<0.001**
FPG (mmol/L)	5.94 (5.32-6.67)	6.23 (5.38-7.27)	7.06 (6.05-8.11)	**<0.001**
FC-P (pmol/L)	540.42 (364.31-765.56)	488.07 (312.21-656.53)	458.26 (282.45-660.08)	**0.038**
HOMA-IR	2.65 (2.26-3.06)	2.51 (2.19-3.13)	2.66 (2.17-3.33)	0.629
HOMA-β	56.47 (36.72-90.11)	47.12 (31.03-72.15)	33.54 (19.13-49.98)	**<0.001**
TG (mmol/L)	1.38 (0.97-2.01)	1.31 (0.94-1.79)	1.29 (0.94-1.79)	0.626
TC (mmol/L)	4.28 (3.63-4.97)	4.37 (3.62-5.10)	4.39 (3.34-5.66)	0.912
HDL-c (mmol/L)	1.08 (0.90-1.28)	1.09 (0.90-1.31)	1.00 (0.85-1.20)	0.117
LDL-c (mmol/L)	2.48 (1.77-2.98)	2.42 (1.85-3.09)	2.30 (1.66-3.51)	0.972
BUN (mmol/L)	5.58 (4.80-6.60)	6.12 (5.05-7.21)	6.44 (5.41-7.58)	**0.004**
eGFR (mL/min/1.73m²)	107.47 (99.69-112.84)	99.93 (93.67-106.08)	94.80 (85.06-99.65)	**<0.001**
Scr (mg/dL)	63.60 (51.40-73.90)	66.00 (56.40-75.05)	69.70 (62.95-82.85)	**<0.001**
UACR (mg/g)	11.45 (7.24-20.12)	12.95 (7.60-24.87)	18.16 (9.79-45.01)	**0.004**
hsCRP (mg/L)	0.60 (0.30-1.80)	0.60 (0.30-1.70)	0.80 (0.40-2.25)	0.186

ADOD, Age at Diagnosis of Diabetes; BMI, body mass index; SBP, systolic blood pressure; DBP, diastolic blood pressure; HbA1c, hemoglobin A1c; GA, Glycated Serum Albumin; FPG, fasting plasma glucose; FC-P, Fasting C-peptide; HOMA-IR, Homeostasis Model Assessment of Insulin Resistance; HOMA-b, Homeostasis Model Assessment of Beta Cell Function; TG, triacylglycerol; TC, total cholesterol; HDL-c, high-density lipoprotein cholesterol; LDL-c, low-density lipoprotein cholesterol; eGFR, estimated glomerular filtration rate; Scr, serum creatinine; BUN, blood urea nitrogen;UACR, urinary albumin/creatinine ratio; hs-CRP, high-sensitivity C-reactive protein. Values in bold denote statistical significance (p < 0.05).

The baseline characteristics of T2DM patients with (332, 87.8%) and without (46, 12.2%) progression in 10-year CVD risk in the SCORE2-Diabetes after COVID-19 infection are shown in **Table 2**. Alcohol consumption, hs-CRP and eGFR were significantly different in the progressed group (*P*<0.05). However, no discrepancies were detected in additional clinical attributes across the groups (**Table 2**).To identify risk factors for 10-year CVD risk progression in T2DM patients post-COVID-19, we conducted both univariate and multivariate logistic regression analyses using key data points from **Table 2** (**Table 3**). Multivariate analysis revealed that alcohol consumption and hs-CRP were positively associated with 10-year CVD risk progression, while eGFR showed a negative association, all with statistical significance (OR 2.10, 95% CI 1.02-4.34; OR 1.33, 95% CI 1.13-1.57; OR 0.96, 95% CI 0.94-0.99).

**Table 2:** Comparison of general information at baseline between progressive and non-progressive T2DM with 10-year CVD risk.

Variable	Non-progression (n = 332, 87.8%)	Progression (n = 46, 12.2%)	*P*-value
Male, n (%)	217 (65.4)	34 (73.9)	0.325
Smoking, n (%)	87 (26.2)	17 (37.0)	0.176
Alcohol consumption, n (%)	104 (31.3)	22 (47.8)	**0.04**
Age (years)	53.00 (46.00-60.00)	55.50 (50.00-60.75)	0.212
ADOD (years)	46.50 (41.00-54.00)	47.50 (43.00-55.00)	0.778
Height (cm)	167.62 ± 8.29	169.34 ± 7.90	0.187
Weight (kg)	69.45 (62.77-78.03)	69.75 (63.45-76.45)	0.747
BMI (kg/m²)	24.70 (22.90-26.70)	23.70 (22.40-26.17)	0.086
SBP (mmHg)	129.00 (118.00-141.00)	129.00 (120.50-136.00)	0.769
DBP (mmHg)	76.87 ± 10.32	74.98 ± 9.32	0.239
HbA1c (%)	6.50 (5.90-7.23)	6.55 (6.00-6.90)	0.374
GA (%)	14.93 (12.80-17.76)	15.11 (13.83-17.51)	0.640
FPG (mmol/L)	6.23 (5.46-7.27)	6.15 (5.32-6.94)	0.370
FC-P (pmol/L)	530.31 (338.46-725.31)	512.80 (323.45-710.41)	0.554
HOMA-IR	2.67 (1.55-3.17)	2.55 (1.43-3.05)	0.170
HOMA-β	48.91 (28.48-77.84)	45.66 (31.09-62.09)	0.493
TG (mmol/L)	1.35 (0.96-1.91)	1.15 (0.86-1.79)	0.160
TC (mmol/L)	4.32 (3.57-5.05)	4.15 (3.61-5.32)	0.736
HDL-c (mmol/L)	1.07 (0.89-1.26)	1.08 (0.91-1.28)	0.682
LDL-c (mmol/L)	2.46 (1.80-3.11)	2.42 (1.62-2.93)	0.693
eGFR (mL/min/1.73m²)	101.70 (94.03-109.50)	97.34 (90.73-103.01)	**<0.001**
Scr (mg/dL)	0.74 ± 0.24	0.78 ± 0.50	0.064
BUN (mmol/L)	5.95 (4.90-7.16)	6.30 (5.72-7.03)	0.120
UACR (mg/g)	13.11 (7.53-26.26)	12.00 (7.23-26.01)	0.701
hs-CRP (mg/L)	0.60 (0.30-1.63)	1.75 (0.52-2.80)	**0.002**

ADOD, Age at Diagnosis of Diabetes; BMI, body mass index; SBP, systolic blood pressure; DBP, diastolic blood pressure; HbA1c, hemoglobin A1c; GA, Glycated Serum Albumin; FPG, fasting plasma glucose; FC-P, Fasting C-peptide; HOMA-IR, Homeostasis Model Assessment of Insulin Resistance; HOMA-b, Homeostasis Model Assessment of Beta Cell Function; TG, triacylglycerol; TC, total cholesterol; HDL-c, high-density lipoprotein cholesterol; LDL-c, low-density lipoprotein cholesterol; eGFR, estimated glomerular filtration rate; Scr, serum creatinine; BUN, blood urea nitrogen;UACR, urinary albumin/creatinine ratio; hs-CRP, high-sensitivity C-reactive protein. Values in bold denote statistical significance (p < 0.05).

**Table 3:** Univariate and multivariate logistic regression analysis of 10-year risk of CVD progression.

Variable	Univariate		Multivariate	
	Odds Ratio (95% CI)	*P*-value	Odds Ratio (95% CI)	*P*-value
Male, n (%)	1.50 (0.75,3.01)	0.252		
Smoking, n (%)	1.65 (0.86,3.15)	0.129	1.36 (0.67,2.77)	0.400
Alcohol consumption, n (%)	2.01 (1.08,3.75)	**0.028**	2.10 (1.02,4.34)	**0.045**
BMI (kg/m²)	0.91 (0.82,1.01)	0.086	0.90 (0.80,1.01)	0.064
Age (years)	1.02 (0.98,1.06)	0.247		
hs-CRP (mg/L)	1.27 (1.11,1.47)	**<0.001**	1.33 (1.13,1.57)	**<0.001**
eGFR (mL/min/1.73m²)	0.96 (0.94,0.98)	**<0.001**	0.96 (0.94,0.99)	**0.002**

CI, Confidence Interval.

BMI, body mass index; hs-CRP, high-sensitivity C-reactive protein; eGFR, estimated glomerular filtration rate.

Values in bold denote statistical significance (p < 0.05).

Additional analyses assessed the link between identified risk factors and 10-year CVD risk progression (**Table 4**). Alcohol consumption (OR, 95%CI: 2.01, 1.08-3.75, *P*=0.028) significantly increased 10-year CVD risk progression in Model 1 (unadjusted), and this correlation persisted (Model 2) post-adjustment for age, sex, smoking status, and BMI (OR, 95%CI: 2.08, 1.01-4.29, *P*=0.047), indicating it as an independent risk factor. Elevated hs-CRP (≥1.0 mg/L) showed significant association with risk progression in both models, though the highest group (>3.0 mg/L) did not demonstrate further increased risk, suggesting potential nonlinearity. In contrast, the highest eGFR quartile group (≥108.43 mL/min/1.73m²) demonstrated a significant protective effect (OR, 95%CI: 0.05, 0.01-0.36, *P*=0.003), which remained stable in Model 2 (OR, 95%CI: 0.05, 0.00-0.29, *P*=0.003).

**Table 4:** Associations of potential risk factors on 10-year CVD risk progression in T2DM patients infected with COVID-19.

Categories	Model 1		Model 2	
	OR (95%CI)	*P*-value	OR (95%CI)	*P*-value
Alcohol consumption				
No	1		1	
Yes	2.01 (1.08,3.75)	**0.028**	2.08 (1.01,4.29)	**0.047**
hs-CRP (mg/L)				
< 1.0	1		1	
1.0~3.0	3.78 (1.81,7.86)	**<0.001**	3.81 (1.83,7.95)	**<0.001**
>3.0	3.25 (1.22,8.66)	**0.018**	3.23 (1.21,8.62)	**0.019**
eGFR (mL/min/1.73m²)				
<92.93 (Q1)	1		1	
92.93~100.88 (Q2)	0.58 (0.25,1.30)	0.183	0.58 (0.26,1.24)	0.194
100.89~108.42 (Q3)	0.65 (0.30,1.48)	0.315	0.65 (0.26,1.26)	0.302
≥108.43 (Q4)	0.05 (0.01,0.36)	**0.003**	0.05 (0.00,0.29)	**0.003**

Model 1: Non-adjusted.

Model 2: Adjusted for age, gender, smoking and BMI.

Values in bold denote statistical significance (p < 0.05).

**Figure 2:**
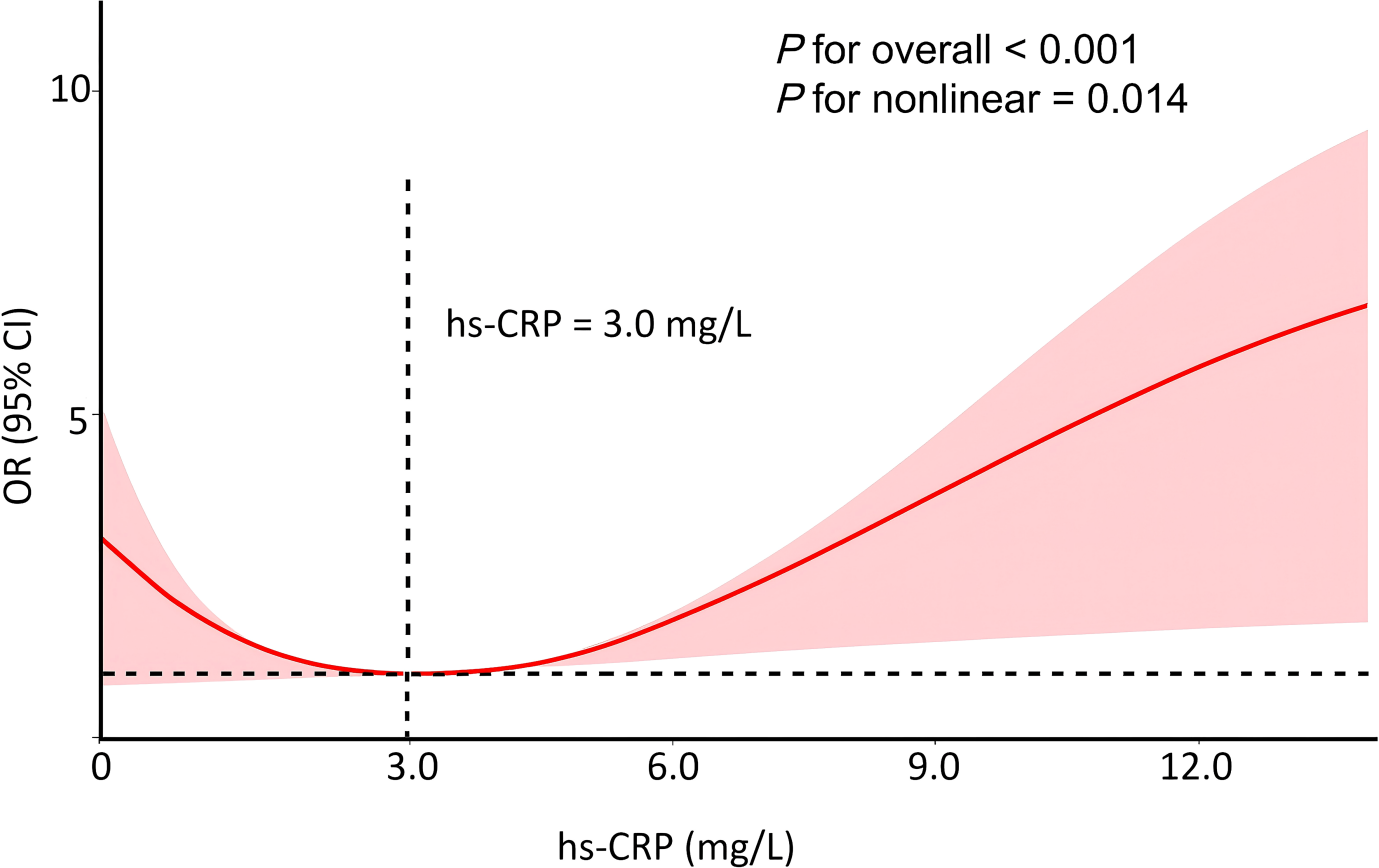
Association between hs-CRP and 10-year CVD risk progression in T2DM patients infected with COVID-19. Adjusted for age, gender, BMI and smoking status.

We used restricted cubic sample (RCS) analysis to test for a nonlinear relationship between hs-CRP levels and 10-year CVD risk progression. After adjusting for potential confounding variables, found a nonlinear relationship between hs-CRP levels and 10-year CVD risk progression in T2DM patients infected with COVID-19 (*P*-overall<0.001, *P*-nonlinear=0.014) (**Figure 2**). The threshold effect analysis pinpointed 3.0 mg/L as the critical hs-CRP value. Below this threshold, every 1 mg/L rise in hs-CRP corresponded to a striking 128% increased risk (OR 2.28, 95% CI 1.48-3.55, *P*<0.001). No statistically meaningful association emerged when hs-CRP levels exceeded 3.0 mg/L (OR: 1.17, 95% CI 0.82-1.66, *P*=0.382), as detailed in **Table 5**.

**Table 5:** Threshold effect analysis of hs-CRP on 10-year CVD risk progression in T2DM patients infected with COVID-19.

	Adjusted OR (95%CI)	*P*-value
Fitting by two piecewise vogistic model		
Inflection point	3.0	
High-sensitivity C reactive protein < 3.0	OR=2.28 (95%CI:1.48-3.55)	**<0.001**
High-sensitivity C reactive protein ≥ 3.0	OR=1.17 (95%CI:0.82-1.66)	0.382
*P* for Log-likelihood ratio		**0.028**

Values in bold denote statistical significance (p < 0.05).

**Figure 3:**
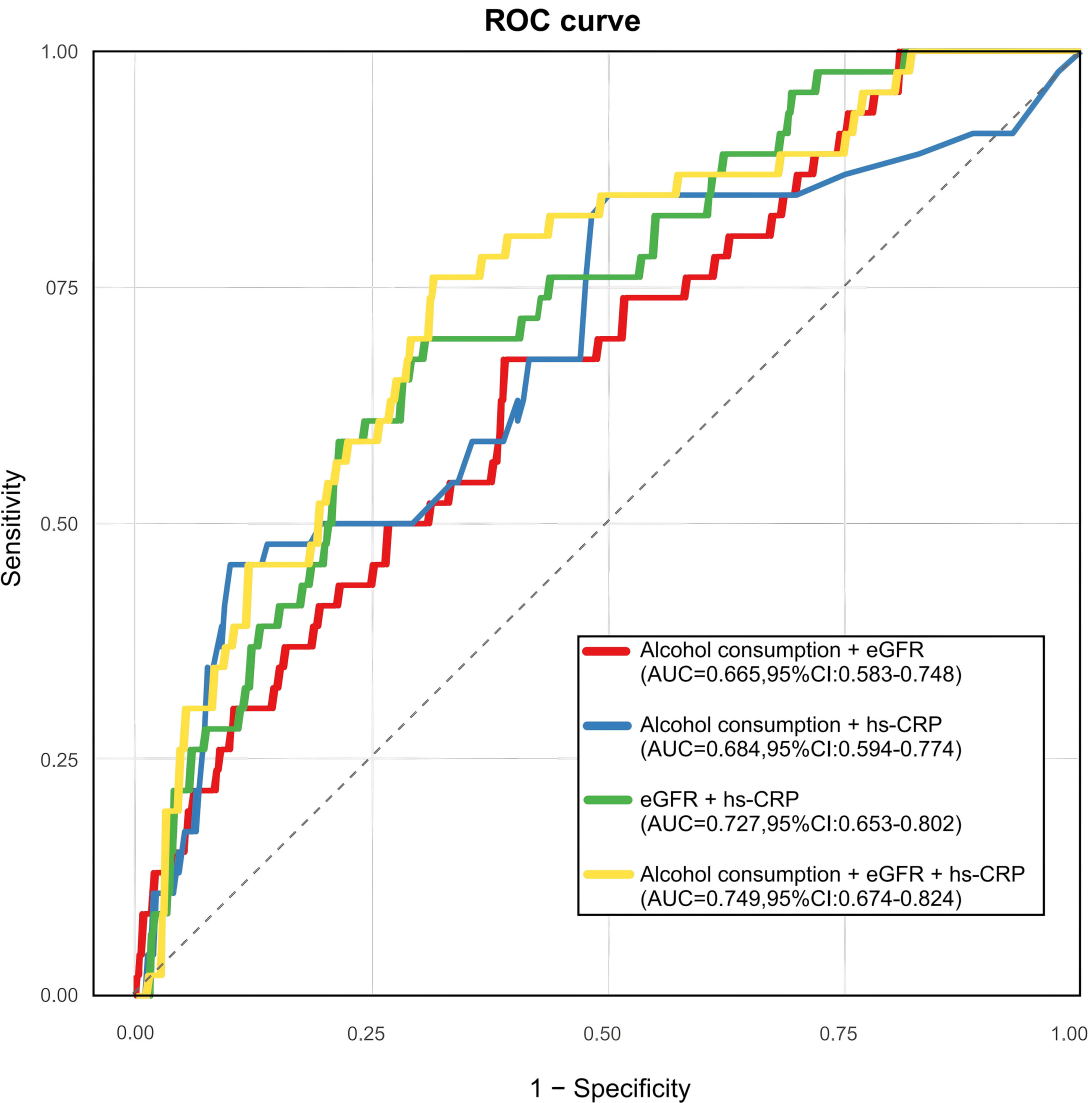
ROC curve of different risk factor combination models. (Model1: Alcohol consumption + eGFR; Model2: Alcohol consumption + hs-CRP; Model3: eGFR + hs-CRP; Model4: Alcohol consumption + eGFR + hs-CRP).

To assess the effect of alcohol consumption, hs-CRP and eGFR in predicting the progression of the 10-year CVD risk in T2DM patients infected with a COVID-19, we constructed receiver operating characteristic (ROC) curves (**Figure 3**). For combined predictors: Model 1 (alcohol consumption + eGFR) and Model 2 (alcohol consumption + hs-CRP) showed similar AUC [0.665 (95%CI: 0.583-0.748) *vs* 0.684 (95%CI: 0.594-0.774)]. Model 3 (eGFR + hs-CRP) improved predictive performance [0.727 (95%CI: 0.653-0.802)], while the full model (alcohol consumption + eGFR + hs-CRP) achieved maximal AUC [0.749 (95%CI: 0.674-0.824)].

## 4 Discussion

In this retrospective cohort analysis, alcohol consumption, hs-CRP levels and eGFR significantly correlated with 10-year CVD risk progression in T2DM patients. Higher hs-CRP levels and alcohol consumption were significantly associated with increased CVD risk, while higher eGFR levels were inversely associated with risk progression.

High-sensitivity C-reactive protein (hs-CRP) serves as a significant inflammatory marker linked to atherosclerotic cardiovascular disease (36–38). Research in a Chinese ischemic stroke cohort linked elevated hs-CRP levels to higher all-cause mortality risk (39). Our study also confirmed elevated hs-CRP correlated with increased CVD risk (OR, 95% CI: 1.33, 1.13-1.57, *P*<0.001). Several studies have linked elevated hs-CRP levels to less favorable clinical outcomes in COVID-19 patients, dictating its potential as a biomarker for predicting clinical severity. This association might be explained by the cytokine storm induced by severe COVID-19, which can worsen insulin resistance (40, 41). Pro-inflammatory agents like tumor necrosis factor-α (TNF-α), interleukin-6 (IL-6), interleukin-1β (IL-1β), and vascular endothelial growth factors can further exacerbate vascular injury and endothelial dysfunction, leading to vascular remodeling (42). IL-6 is a potent stimulator of CRP production, produced by vascular smooth muscle cells in reaction to atherosclerosis. CRP binds directly to severely atherosclerotic oxidized LDL-C and is found in lipid-rich plaques (43). CRP contributes to plaque development through complex proatherogenic interactions with various atherogenic cells (44). CRP potentially fosters monocyte attachment and movement within vessel walls, a key early phase of atherosclerosis (45). Furthermore, CRP-catalyzed M1 macrophage polarization acts as a pro-inflammatory initiator in plaque development, promoting macrophage accumulation in adipose tissue and atherosclerotic plaques (46). Monomeric CRP, an isoform activated by platelets, exhibits distinct prothrombotic and inflammatory characteristics (47), and it’s also occurs within plaques, notably regions displaying monocyte-driven inflammation, as well as in the lipid microstructural domains of endothelial cells (48). Clinical trials have examined hs-CRP thresholds of both 2 mg/L and 3 mg/L to ascertain heightened CVD risk (37, 49). Research consistently shows a marked link between cardiovascular incidents and hs-CRP levels exceeding 3 mg/L, when contrasted with those below 1 mg/L (50–52), the Multi-Ethnic Study of Atherosclerosis (MESA) reported a 50% increased risk at the 2 mg/L threshold (50–52). Specifically, this subjects showed a mean hs-CRP of 3.76 mg/L and with notably elevated levels in those who had future coronary events and those who did not (53). This discrepancy may stem from population differences in inflammatory baseline levels or distinct CVD risk stratification methods. In contrast, our study identified 3.0 mg/L as a potential inflection point. Differently, when in the range of hs-CRP <3.0 mg/L, for every 1 mg/L rise in hs-CRP, the likelihood of a 10-year CVD risk escalation grew by 128% (OR, 95% CI: 2.28, 1.48-3.55, *P*<0.001), with high statistical significance, suggesting that hs-CRP is a key driver of CVD risk at low inflammation levels. When hs-CRP was ≥3.0 mg/L, the hs-CRP level was still positively correlated with CVD risk (OR=1.17), but this correlation was not statistically significant. This attenuation may reflect reduced statistical power due to a smaller subgroup size, or it may suggest a more complex risk landscape in high-inflammatory states where other pathological mechanisms coexist. Inflammation control may be particularly critical in the lower hs-CRP range, while a multifactorial approach may be warranted in individuals with elevated hs-CRP. This observation warrants further investigation, including studies employing non-linear modeling and biological validation to assess the potential existence and clinical relevance of such thresholds.

The cardiovascular-kidney-metabolic (CKM) syndrome concept highlights the interconnected nature of T2DM, CKD, and CVD. T2DM, as a core driver of metabolic abnormalities, directly damages the glomerular basement membrane integrity and vascular endothelium activity, leading to the synergistic progression of CKD and atherosclerotic CVD (54). The COVID-19 pandemic exacerbates renal function decline in patients with T2DM through direct infection of renal tubular cells via the ACE2 receptor, as well as the exacerbation of podocyte injury by hyperglycemia, leading to decreased eGFR and increased proteinuria (55). In addition, COVID-19-induced cytokine storm and oxidative stress, synergized with the pathomechanisms of diabetic nephropathy, accelerate glomerulosclerosis and eGFR decline (56). Bowe et al. (57) demonstrated that COVID-19 survivors exhibited higher rates of acute kidney injury (AKI), eGFR decline, end-stage renal disease (ESKD), major adverse renal events (MAKE), and a steeper longitudinal decline in eGFR within 30 days post-acute phase. This renal impairment induced by COVID-19 may further amplify the established association between renal dysfunction and cardiovascular outcomes. eGFR’s association with CVD risk exhibits non-linearity, with both low eGFR and glomerular hyperfiltration increasing CVD risk (58). This non-linear relationship may be explained by the fact that glomerular hyperfiltration reflects glomerular hypertension, while low filtration reflects impaired excretory function. The Second National Health and Nutrition Examination Survey (NHANES II) (59) indicated that patients with an eGFR below 70 mL/min/1.73 m² faced a 68% heightened risk of overall mortality and a 51% greater risk of cardiovascular death versus those with an eGFR of 90 mL/min/1.73 m² or higher. The Atherosclerosis Risk in Communities Study (ARIC) (60) showed that individuals with baseline eGFR in the range of 15–59 mL/min/1.73 m² had a 38% elevated risk of cardiovascular disease compared to the eGFR 90–150 mL/min/1.73 m² group. Furthermore, Seung et al. (61) reported that glomerular hyperfiltration was associated with CVD, especially myocardial infarction and heart failure. In our study, after infection with COVID-19 in T2DM patients, the baseline level of eGFR was significantly lower in the population with a progressive 10-year CVD risk than in the non-progressive population (*P*<0.001). In multivariate stepwise logistic regression analyses, higher eGFR levels were significantly associated with lower odds of 10-year CVD risk progression (OR, 95%CI: 0.96, 0.94-0.99, *P*=0.002). The inverse association was most pronounced in the highest quartile of eGFR (≥108.43 mL/min/1.73m²), where participants had substantially lower odds of risk progression (OR, 95%CI: 0.05, 0.01-0.36, *P*=0.003). This association remained consistent after adjustment for age, sex, BMI, and smoking in Model 2 (OR, 95%CI: 0.05, 0.00-0.29, *P*=0.003). A comprehensive meta-analysis encompassing 12 cohort studies (62) revealed that participants with an eGFR between 75–105 mL/min/1.73m² exhibited the lowest risk of all-cause and CVD mortality. Conversely, those with an eGFR below 60 mL/min/1.73m²faced a nearly twofold higher mortality rate (*P*<0.001). From the CKM perspective, T2DM, CKD, and CVD are tightly intertwined. Maintaining optimal eGFR may be a key strategy to delay the progression of CKM syndrome, and a combination of multidisciplinary interventions will be needed to improve patients’ prognosis in the future.

Numerous studies have demonstrated a double-edged relationship between alcohol consumption and cardiovascular disease. Alcohol is associated with a well-documented J-shaped dose-effect curve, with mild to moderate alcohol consumption reducing cardiovascular and total mortality (63–65), while excessive or binge drinking has the opposite effect (64, 66). This may be an amplified effect of cytokine storm in T2DM patients infected with COVID-19. Wang et al. (67) showed that the alcohol metabolite acetaldehyde activates the NF-κB pathway and promotes the release of pro-inflammatory factors, such as IL-6 and TNF-α, which superimposes with the SARS-CoV-2-induced cytokine storm, thus exacerbating CVD risk in patients with T2DM in terms of inflammatory pathways. In the analysis of our research, the link between drinking alcohol and the progression of CVD over a 10-year period held strong even when we accounted for other factors that might skew the results (OR 2.08, 95% CI 1.01-4.29). This indicates that alcohol intake is a standalone predictor for 10-year CVD risk for T2DM patients. Chronic hyperglycemia and insulin resistance in T2DM trigger excessive production of reactive oxygen species (ROS), triggering thrombosis, inflammation, imbalance of vascular homeostasis, and cell proliferation, and several cardiovascular risk factors correlate with elevated ROS generation or reduced plasma glutathione (GSH) levels (68, 69). GSH, a mitochondrial bio-antioxidant in mammalian tissues, plays a key role in reducing oxidative stress. SARS-CoV-2 reduces intracellular GSH levels by impairing NRF2 activity, a key regulator of oxidative defense that enhances GSH synthesis (70, 71). Studies have shown that alcohol can exacerbate oxidative stress in COVID-19 patients by depleting GSH through the dual pathway of inhibiting glutathione synthase (GCL) activity and promoting GSH oxidation (72). Overall, alcohol consumption significantly amplifies CVD risk in T2DM patients following COVID-19 infection by activating inflammatory pathways (e.g., NF-κB/IL-6 axis) and exacerbating oxidative stress (GSH depletion). In our study, patients who drank alcohol weekly or almost weekly were defined as having a drinking habit, but only frequency was recorded, failing to differentiate on specific amounts of alcohol consumed, which is something that might be further investigated through detailed research to delve into the dual impact of alcohol on heart disease risk. Future studies need to further resolve the molecular mechanisms of alcohol-virus synergism and incorporate lifestyle interventions such as alcohol restriction to optimize cardiovascular risk management in the T2DM population.

Several salient strengths warrant mention in the present study. Employing novel methodological integration, we present the first-ever systematic analysis of 10-year CVD predictors among individuals with type 2 diabetes during the COVID-19 era, integrated with the SCORE2-Diabetes model, providing new evidence for the management of CKM syndrome. Secondly, the study used multifactorial regression, nonlinear analysis and threshold effect validation, which made the results more reliable. Finally, we also suggested that the joint prediction model of alcohol consumption, eGFR and hs-CRP (AUC=0.749) has potential clinical applications. In clinical risk prediction literature, the area under the receiver operating characteristic curve (AUC) between 0.7 and 0.8 is typically regarded as indicative of acceptable to good discriminatory ability, whereas values exceeding 0.8 denote excellent performance. Consequently, our model’s AUC of 0.749 suggests a moderate, yet clinically meaningful, level of discrimination. This is particularly relevant within the context of post-COVID-19 cardiovascular risk stratification in patients with type 2 diabetes. While not definitive for diagnostic purposes, this level of performance supports the model’s potential value as a supplementary tool in early screening and risk-based intervention planning.

## 5 Limitations

This research is not without its shortcomings. For starters, the modest participant pool could compromise the reliability of our statistical findings, and with the relatively small sample size of the progression group, the observed heterogeneity between groups may affect the robustness of the regression model. Additionally, since all data was collected from a single institution, the results may be skewed by selection bias and lack broader applicability. Third, retrospective study designs have inherent limitations in causal inference, with the inability to infer causality and the possibility of selection bias and unmeasured confounders, such as statin use, the severity of COVID-19 and COVID-19 vaccination status. Statins are widely prescribed in patients with diabetes and are known to significantly reduce cardiovascular events (73). The absence of statin use data may have led to underestimation of CVD risk in some individuals. COVID-19 severity may affect long-term cardiovascular outcomes by increasing inflammation, endothelial dysfunction, and myocardial injury (19). The lack of COVID-19 severity grading may have obscured the association between inflammation and CVD risk progression. Vaccinated individuals have been shown to exhibit 10–27% lower rates of cardiovascular events following infection, compared to unvaccinated counterparts (74). This omission may result in residual confounding by vaccination. These variables should be included in future prospective studies to further improve the accuracy of CVD risk assessment in T2DM patients following COVID-19 infection. Fourth, the lack of data on long-term post-infection follow-up does not allow assessment of the long-term impact of 10-year CVD risk progression in T2DM patients, and dynamic changes in risk need to be tracked through multicenter prospective cohorts. Fifth, alcohol consumption was assessed by frequency in this study, without accounting for volume or drinking patterns, which may reduce the interpretability of the findings, future research should consider employing validated tools such as Alcohol Use Disorders Identification Test–Consumption (AUDIT-C) for more comprehensive alcohol behavior assessment.

## 6 Conclusion

In this study, we investigated the predictors associated with a progression in the SCORE2-Diabetes score in individuals with type 2 diabetes infected with COVID-19. The results showed that alcohol consumption and elevated hs-CRP levels were significantly associated with progression of 10-year CVD risk, while higher eGFR levels were inversely associated with risk progression. These findings highlight potential associations that may inform future research on inflammation control, alcohol-related behaviors, and renal function in cardiovascular risk management. Despite the limitations of the retrospective study, the results also contribute to the clinical application of CKM syndrome management. Elevated hs-CRP reflects residual inflammatory risk; reduced eGFR indicates early renal vulnerability; and alcohol use represents a modifiable behavioral driver of metabolic dysregulation. Together, these markers enable integrated risk stratification across cardiovascular, renal, and metabolic axes. Incorporating them into routine assessment allows early identification of high-risk CKM phenotypes and supports the implementation of targeted, dimension-specific interventions, thereby advancing the goal of point-to-point CKM management.

## Data Availability

The datasets presented in this article are not readily available because The data that support the findings of this study are available from Metabolic Management Center (MMC) of Shanghai First People’s Hospital but restrictions apply to the availability of these data, which were used under license for the current study, and so are not publicly available. Data are however available from the authors upon reasonable request and with permission of Metabolic Management Center (MMC) of Shanghai First People’s Hospital. Requests to access the datasets should be directed to wangyizhe5568@163.com.
